# Cancer Informatics Vision: caBIG™

**Published:** 2007-02-06

**Authors:** Andrew C. von Eschenbach, Kenneth Buetow

**Affiliations:** 1 Director, National Cancer Institute; 2 Director, National Cancer Institute Center for Bioinformatics, Bethesda, MD

The National Cancer Institute (NCI) plays a critically important role in providing vision and leadership for cancer research in the United States and around the world. Three decades ago the United States government recognized the importance of mobilizing an effort against cancer and passed the National Cancer Act. Since that time we have seen the success of transforming scientific discoveries into treatments, illustrated by the fact that today there are over 10 million cancer survivors.

We are in the midst of an explosion of knowledge about cancer as a disease process. We are beginning to understand cancer not by what we can see and touch – or by what is revealed under a microscope – but at the molecular level. It is not a question of if, but rather when and how, molecular medicine translates into personalized care. As we understand more completely the steps of the cancer process, we will identify the specific molecular targets in that process that are vulnerable to preemption. Based upon the realization of how much can be accomplished by coming together as teams and integrating our robust biomedical research enterprise, NCI has challenged the oncology community to eliminate the suffering and death due to cancer by 2015.

We cannot achieve this ambitious goal without greater interconnectivity and coordination across the cancer enterprise. This requires seeing cancer as a systems problem that will require a systems solution. Although cancer is being unraveled rapidly at the genomic and proteomic levels, we have not concomitantly developed the seamless system needed to capitalize on our discoveries.

To universally integrate personalized medicine into cancer prevention, diagnosis and treatment, researchers and clinicians must be able to gain rapid access to multiple types of specific information about an individual patient—information to which they do not currently have easy access. A new generation of medicine will require incorporation of shared information technologies (IT).

The information infrastructure revolution that has transformed business has had slow uptake in biology and medicine. NCI is committed to creating a standards-based biomedical infrastructure and to making it available across the entire cancer enterprise. In 2003, NCI launched the cancer Biomedical Informatics Grid (caBIG™), an international collaboration to facilitate and enable research teams to share data, applications, and infrastructure that facilitate collaborations that accelerate the conversion of data from information to knowledge.

Within the cancer research community there exists a “Tower of Babel” problem. Research teams cannot easily understand data collected by—or share data with—other medical research teams working on the very same disease. Efficient, effective collaborations are blocked by these “language” and data sharing problems. Scientists have a difficult time integrating the various types of data they collect in a manner that will allow them to ask and answer important questions about how a disease works, and what they can do to stop it.

Medical research teams have operated, in effect, as cottage industries, each collecting and interpreting data using a unique language of their own making and in virtual isolation from other teams. Biomedical informatics has the potential to be the powerful critical means to achieve the necessary degree of integration as it provides the mechanisms and tools to support standardized sharing, management and analysis of diverse data across the bench-to-bedside continuum and back.

Recognizing the transformational power of an interoperable biomedical informatics infrastructure to overcome these obstacles, caBIG™ is constructed around three interrelated areas of informatics:

- Bioinformatics provides cancer and biomedical researchers with tools, infrastructure and analytic methodologies necessary to manage and harvest insights from the large volumes of data generated by novel types of research such as molecular biology, genomics and proteomics.
- Medical or clinical informatics enables the management, analysis and dissemination of clinical and public health data, and includes the use of informatics infrastructure and applications such as clinical trial management systems, electronic health records, and cancer registries.
- Biomedical informatics, an innovative synergy between bioinformatics and clinical informatics, offers infrastructure, tools, techniques and applications that bridge the two other areas and creates a mechanism that facilitates the sharing of data along the continuum from research bench to clinical bedside and back. It offers the prospect of integrating individual patient data from clinical care into the clinical research environment, and back into clinical care or basic science research.

Utilizing these principles, caBIG™ is being created by the cancer research community, for the cancer research community. caBIG™’s software engineers are developing standardized data collection and data management tools that will allow laboratory and clinical researchers to integrate their own data more effectively, as well as to share data more easily with others. In addition, they are developing data analysis tools that will improve the speed and productivity of basic cancer research and clinical trials being conducted in the cancer community across the country, and around the world. In short, they are developing a “fully interoperable” IT network that will supercharge the entire field of cancer research.

**Figure:**
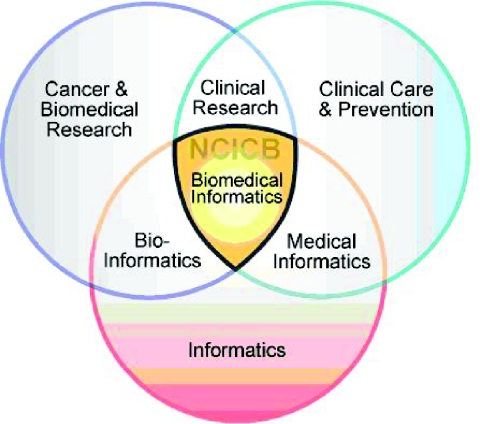


caBIG™ is being developed specifically to enable and accelerate the “bench-to-bedside-and-back” cycle. It is a network of information and tools that will allow cancer researchers, clinicians, and patients to easily share, integrate, and analyze data from many different sources. Enabling seamless connections between the distinct areas of research and between the many necessary sources of information will facilitate the creation of a new era of faster, and more effective personalized care by interdisciplinary teams of cancer researchers and clinicians.

caBIG™ is being built on open source, open access, open development, and federation principles. Anyone can gain access to caBIG™ software (and its component parts) at no cost, modify it to suit his or her needs, and contribute to its ongoing development. Many organizations are working together to realize its full potential, including for-profit companies that wish to “add value” to caBIG™ – i.e., enhance caBIG™’s function or ease of use – and lease or sell those improvements to interested caBIG™ users.

As an open source model, source code for all caBIG™ software tools is available to end-users. This approach is consistent with the philosophy supporting development of the “knowledge commons,” which is created and fed by free and open distribution of intellectual property on the internet. We share the goal of the Science Commons, to encourage scientific innovation by making it easier for scientists, universities, and industries to use literature, data, and other scientific intellectual property, and to share their knowledge with others. As is the case with the Science Commons, a recently established project of the on-line conservancy service known as the Creative Commons, caBIG™ will be engineered to function within current copyright and patent law to promote legal and technical mechanisms that remove barriers to sharing.

As the transforming power of an integrated infrastructure becomes more widely appreciated, an ever increasing number of stakeholders are examining their own environments and asking how they can build interconnecting links through caBIG™. The foundational components of caBIG™ are readily available as building blocks, connectors, and tools. These components have broad utility beyond the cancer community, because much of the information and processes associated with cancer prevention, care, and research share fundamental elements or approaches with other health challenges.

Sharing and integrating functional genomics and clinical trial data can improve cancer prevention and treatment beyond any one country. The burden of cancer is international. caBIG™ is collaborating with the National Cancer Research Institute in the United Kingdom to enable a richer analysis of complex relationships between, for example, patterns of gene expression and prognosis or response to treatment.

When appropriately delivered and used, biomedical informatics will provide an interconnected web of information and tools that link together the multitude of participants involved in the cancer endeavor. Although caBIG™’s most important accomplishments and contributions still lie in the future, it has the power to redefine how cancer research is conducted and shared. caBIG™ is hastening the time when patients live with, rather than die from cancer.

## Related web pages

**caBIG:** https://cabig.nci.nih.gov/

**NCICB:** http://ncicb.nci.nih.gov/

**NCRI:** http://www.ncri.org.uk/

**Science Commons:** http://sciencecommons.org/

